# Oral and Gingival Crevicular Fluid Biomarkers for Jawbone Turnover Diseases: A Scoping Review

**DOI:** 10.3390/diagnostics14192184

**Published:** 2024-09-30

**Authors:** Nurfatima Azzahra Fadli, Mariati Abdul Rahman, Saiful Anuar Karsani, Roszalina Ramli

**Affiliations:** 1 Department of Craniofacial Diagnostics and Biosciences, Faculty of Dentistry, Universiti Kebangsaan Malaysia, Jalan Raja Muda Abdul Aziz, Kuala Lumpur 50300, Malaysia; nurfatimafadli@gmail.com; 2 Institute of Biological Sciences, Faculty of Science, Universiti Malaya, Kuala Lumpur 50603, Malaysia; saiful72@um.edu.my; 3 Department of Oral and Maxillofacial Surgery, Faculty of Dentistry, Universiti Kebangsaan Malaysia, Kuala Lumpur 50300, Malaysia

**Keywords:** biomarkers, bone turnover diseases, jawbones, oral fluid, gingival crevicular fluid, saliva

## Abstract

Gingival crevicular fluid (GCF) and oral fluid have emerged as promising diagnostic tools for detecting biomarkers. This review aimed to evaluate the existing literature on using oral fluids as a source of biomarkers for bone turnover diseases affecting the jawbone. A comprehensive search strategy was executed between August 2014 and August 2024 across five major databases (Web of Science, EBSCOhost Dentistry & Oral Sciences Source, Cochrane Library, Scopus, and PubMed) and grey literature sources. The Preferred Reporting Items for Systematic Reviews and Meta-Analyses extension for Scoping Reviews (PRISMA-ScR) was applied. The screening was facilitated using Rayyan at rayyan.ai and Endnote X20 software tools, culminating in the evaluation of 14,965 citations from databases and 34 from grey literature. Following rigorous scrutiny, 37 articles were selected for inclusion in this review, encompassing diseases such as periodontitis, medication-related osteonecrosis of the jaw (MRONJ), and osteoporosis. The quality of the included observational studies was assessed using the Revised Risk of Bias Assessment Tool for Non-Randomized Studies (RoBANS 2). Interleukin-1 beta (IL-1β), sclerostin, osteoprotegerin (OPG), and interleukin-34 (IL-34) emerged as significant biomarkers in GCF, and they were mainly from periodontitis and osteoporosis. Osteocalcin (OC), IL-1β, tumor necrosis factor-alpha (TNF-α), interleukin-6 (IL-6), OPG, and matrix metalloproteinase-9 (MMP-9) were significant in oral fluid or saliva, and they were from periodontitis, MRONJ, and osteoporosis. These findings underscore the potential use of oral fluids, which are regarded as non-invasive tools for biomarker identification in bone turnover. Many biomarkers overlap, and it is important to identify other specific biomarkers to enable accurate diagnosis of these conditions.

## 1. Introduction

Bone turnover markers can be utilized to assess bone remodeling processes involving osteoclasts and osteoblasts in bone formation and resorption [1]. Generally, bone turnover markers are used to determine bone health and detect individuals at risk of bone-related diseases, particularly osteoporosis [2]. Apart from osteoporosis, bone turnover diseases include bone diseases from endocrine, oncologic, and rheumatologic disorders [3]. In the jawbone, osteoporosis, medication-related osteonecrosis of the jaw (MRONJ), and periodontitis are described as bone turnover diseases [4,5]. The availability of bone turnover markers in oral fluids is limited for medication-related osteonecrosis of the jaw (MRONJ) compared to periodontitis. In bisphosphonate-related osteonecrosis of the jaw (BRONJ), now termed MRONJ, the c-terminal telopeptide cross-link of type I collagen (CTX) has been identified as a bone turnover marker [6]. In osteoporosis, pro-inflammatory cytokines, such as IL-1, IL-6, IL-1β, and TNF-α, have been positively correlated with periodontal bone loss [7]. Bone turnover biomarkers in periodontitis include alkaline phosphatase (AP), osteocalcin (OC), osteoprotegerin (OPG), and receptor activator of nuclear factor kappa beta ligand (RANKL), among others [8].

Standard diagnostic body fluids for biological markers include blood (serum and plasma), excretory products of the body (such as urine, sputum, and saliva), and tissue sections [9,10,11]. Oral fluid is categorized as an oral mucosal transudate (OMT) and saliva [12] while gingival crevicular fluid (GCF) is an inflammatory exudate from the periodontal tissues [13]. Recent studies highlight growing interest in using GCF and saliva biomarkers for early disease detection due to their clinical potential [13,14,15,16,17].

Gingival crevicular fluid (GCF) contains various substances, including cytokines, antibodies, enzymes, and tissue breakdown products [18,19]. The method for collecting GCF involves using filter paper or perio paper, which is inserted into the gingival crevice to absorb the fluid [20,21]. GCF can also be collected through other methods, such as gingival washings, platinum loops, and micropipettes, as described by Sagar et al. [18]. Barros et al. reported that GCF is derived from blood [19], suggesting that GCF sampling could provide results similar to those obtained from blood samples. Biomarkers detected in GCF include matrix metalloproteinase-8 (MMP-8), IL-1β, and IL-6, which were positively associated with increased pocket depth and inflammation of the gums [13]. TNF-α and IL-1β levels were shown to have an association with periodontitis [16,22]. Additionally, these biomarkers are important in the bone remodeling process and can be regarded as a bone turnover marker [23,24,25].

Saliva is a biological fluid that contains numerous molecular components that can serve as biomarkers for diseases [26]. These components include epithelial cells, microorganisms (oral microbiome), and food residues [27]. As a filtrate of blood, saliva also contains components found in blood [28]. Beyond its roles in mastication and lubrication, saliva is essential for swallowing, digestion, and providing protection against oral infections [29]. Saliva sample collection can be performed using four common methods: spitting, swabbing, suction, and draining [30]. Unstimulated saliva is typically collected by having subjects expectorate into a sample tube [21]. Stimulated saliva is usually not used because it leads to more variation in the analysis results compared to unstimulated saliva. The passive drooling method is considered the most accurate collection method with minimal error [30]. Due to the non-invasive nature of saliva collection, diagnostic kits using saliva are frequently employed to detect various conditions, including oral diseases, diabetes, and renal disease [27]. As a result, saliva diagnostic kits are in high demand among consumers for their ease of use, convenience, and rapid results [27].

Bone turnover biomarkers have been proven as useful markers to investigate various diseases [31,32,33]. Some of these biomarkers have been successfully detected through the analysis of GCF and saliva samples, and have been applied in clinical practice [34].

There is abundant literature focusing on the identification of bone turnover markers for oral conditions or diseases using blood and serum. However, studies using oral fluid to identify bone turnover markers are limited. A brief preliminary search using the PubMed, EBSCOhost Dentistry & Oral Sciences Source, and Web of Science databases revealed that numerous studies have been published to identify biomarkers from GCF and saliva separately. The primary objective of this scoping review was to identify bone turnover biomarkers in oral fluids for specific bone turnover diseases affecting the jawbone.

The secondary objective was to elucidate the similarities or differences among the biomarkers in the selected diseases. By gathering the relevant information, this review can enhance our understanding of the available biomarkers and assist researchers in planning their studies.

## 2. Methods

This scoping review was constructed based on the Joanna Briggs Institute (JBI) manual for scoping review and the report was based on the Preferred Reporting Items for Systematic Reviews and Meta-Analyses extension for Scoping Reviews (PRISMA-ScR) [35]. This scoping review was registered in the Open Science Framework (OSF) (https://doi.org/10.17605/OSF.IO/2HAEF) on 20 September 2024. The research questions for this review were as follows: (i) What are the bone turnover biomarkers that can be collected from the oral fluid and GCF of patients who were diagnosed with osteoporosis, MRONJ, and periodontitis? (ii) What are the similarities or differences in the biomarkers in the above diseases?

### 2.1. Eligibility Criteria

Studies were included if they (i) investigated bone turnover biomarkers of jawbone diseases, i.e., osteoporosis, MRONJ, and periodontitis using oral fluid and GCF; (ii) were published in the English language; (iii) the full text of the article was available; and (iv) the type of study included randomized controlled trial study, case-control study, analytical observational study, such as prospective and retrospective cohort studies, or cross-sectional and case series studies. The minimum number of participants in the studies should be ten for cases and ten for controls.

Studies were excluded if they were systematic reviews, case reports, commentaries, editorials, and letters to editors.

### 2.2. Search Strategy

Journal articles were searched using five databases: Web of Science, EBSCOhost Dentistry & Oral Sciences Source, Cochrane Library, Scopus, and PubMed, with pre-discussed search strings. Grey literature, such as Google Scholar, was also explored for additional relevant papers. The search string used was “(Biomarker OR biological marker OR biochemical marker OR biological factor OR clinical marker OR molecular marker) AND (Oral fluid OR fluid and secretion OR gingival crevicular fluid OR saliva OR bodily secretion OR exudate and transudate) AND (Bone disease OR bisphosphonate-related osteonecrosis of the jaw OR medication-related osteonecrosis of the jaw OR osteoporosis OR periodontitis OR periodontal disease)”. Articles were included based on publication year, title, and research design, with further screening based on title, keywords, abstract, and full text.

### 2.3. Study or Source of Evidence Selection

**Figure 1:**
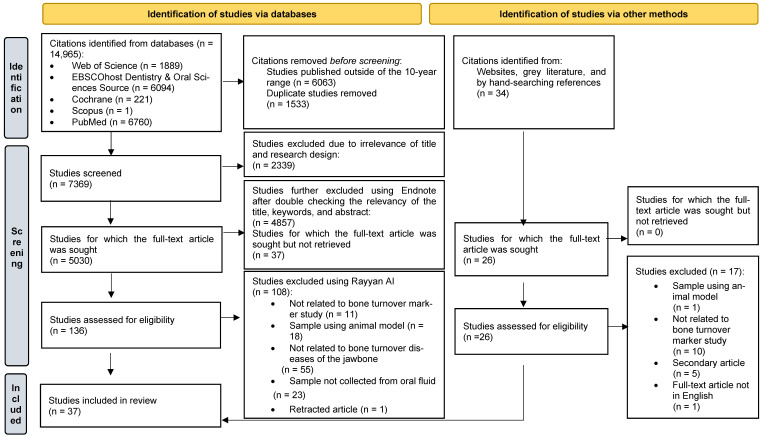
PRISMA flow diagram of the results of the literature search and the selection criteria.

Search results from the five databases were filtered to include publications from August 2014 to August 2024. All citations were imported into Endnote X20 and Rayyan.ai for screening. Duplicates were identified and removed. Two independent reviewers screened the titles, keywords, and abstracts, with full-text articles reviewed when needed. Disagreements were resolved through consensus or with an additional reviewer. The search results, along with reasons for article inclusion and exclusion, were reported in a PRISMA-ScR flow diagram (Figure 1).

### 2.4. Data Extraction

Information from the journal articles was extracted and is summarized in Table 1, including study type, number of subjects, characteristics of cases and controls, oral condition or disease details, assessment tools, and bone turnover markers found in GCF and saliva. A draft extraction form titled “Scoping Review Keywords & Database Search Strategy” is provided in Supplementary S1 to streamline the process of extracting and screening articles. The form includes key search terms, synonyms, search strings, the time and date of the searches, and the number of hits. The final screening results are presented in a table for reference. The form was continuously revised during the review process.

### 2.5. Quality Assessment

The quality of the included observational studies was assessed using the Revised Risk of Bias Assessment Tool for Non-Randomized Studies (RoBANS 2) [36]. This tool evaluates seven domains, assigning a risk of bias as “high”, “low”, or “unclear” to each domain. The seven items were (i) comparability of the target group, (ii) target group selection, (iii) confounders, (iv) measurement of intervention/exposure, (v) blinding of assessors, (vi) outcome assessment, and (vii) incomplete outcome data. The RoBANS 2 tool is suitable for assessing the risk of bias in the results of non-randomized studies, including cohort, case-control, cross-sectional, and before and after intervention studies [36].

**Table 1:** List of studies included in the review.

Author and Year	Type of Study	Number of Subjects and Description of Control(s) and Case(s)	Oral Condition/Disease	Assessment Tools	Expression of Bone Turnover Marker(s) Levels in GCF	Expression of Bone Turnover Marker(s) Levels in Oral Fluid/Saliva
Mustafa et al., 2024 [37]	Cross-sectional study	Number of subjects: 50 male participants aged 50 years or older. Control: Healthy males or males with no osteoporosis * Case: Males with osteoporosis * * Both groups were then divided into asymptomatic and symptomatic patients following temporomandibular disorder (TMD) signs and symptoms.	Osteoporosis	Enzyme-linked immunosorbent assay (ELISA)	-	Calcium ↑ Osteocalcin (OC) ↑ Alkaline phosphatase (ALP) ↑
Sangappa et al., 2024 [38]	Cross-sectional study	Number of subjects: 240 participants with 104 males and 136 females aged 30–69 years old with at least 15 natural teeth (60 per group, four groups in total). Controls: (i) Subjects with no history of Type 2 Diabetes Mellitus (T2DM), not taking systemic drug medication, and glycemic (HbA1c) levels below or equal to 6.4%, and (ii) subjects with chronic periodontitis (CP) only. Cases: (i) Subjects with T2DM (confirmed by HbA1c levels more than or equal to 6.5%) and with CP, and (ii) subjects with T2DM, CP, and tooth loss resulting from periodontitis.	Chronic periodontitis in Type 2 Diabetes Mellitus patients	ELISA	-	Interleukin-6 (IL-6) ↑
Ashifa et al., 2023 [39]	Clinico-biochemical cross-sectional study	Number of subjects: 30 participants with 16 males and 14 females aged 19–40 years old. Control: Subjects with good periodontal health. Cases: (i) Subjects with stage III grade C generalized periodontitis, and (ii) subjects with stage III grade B generalized periodontitis.	Periodontitis	Sandwich ELISA	Sclerostin ↑	-
Gür et al., 2023 [40]	Prospective case-control study	Number of subjects: 71 individuals (34 males and 37 females aged 27–46 years old). Control: 24 subjects with good periodontal health. Cases: (i) 23 subjects with generalized stage III grade C periodontitis, and (ii) 24 subjects with gingivitis.	Periodontitis	ELISA	Periodontal ligament-associated protein-1 (PLAP-1) ↑ Sclerostin ↑ Tumor necrosis factor-alpha (TNF-α) ↑	-
Lorenzo-Pouso et al., 2023 [41]	Case-control study	Number of subjects: 38 male and female subjects. Controls: (i) 10 healthy subjects and (ii) 10 age and gender-matched subjects taking bone-modifying agents (BMAs) for more than 2 years without a diagnosis of MRONJ. Case: 18 MRONJ patients.	Medication-related osteonecrosis of the jaw	Quadrupole–TOF mass spectrometer working in ESI and DDA analysis	-	Matrix metalloproteinase-9 (MMP-9) ↑ Alpha-1-antichymotrypsin (AACT) ↑ Hemoglobin subunit delta (HBD) ↑
Nirubama et al., 2023 [2]	Case-control study	Number of subjects: 80 male and female patients aged 45–60 years old. Control: 40 healthy patients with no sign of osteoporosis. Case: 40 patients with osteoporosis.	Osteoporosis	ELISA	-	Bone-specific alkaline phosphatase (BAP) ↑ Osteocalcin (OC) ↑ C-terminal telopeptide (CTX) ↑ N-terminal telopeptide of type 1 collagen (NTX) ↑
Relvas et al., 2023 [42]	Exploratory observational study	Number of subjects: 68 (21 male and 47 female subjects) individuals aged between 18 and 70 years old with a minimum of 18 natural teeth. Control: 22 patients with good periodontal health. Cases: (i) 17 patients with stage I/II periodontitis, and (ii) 29 patients with stage III/IV periodontitis.	Periodontitis	Multiplex flow assay	-	Interleukin-1β (IL-1β) ↑ RANKL ↑
Renjith et al., 2023 [43]	Case-control study	Number of subjects: 60 patients (33 males and 27 females aged between 32 and 58 years old). Control: 30 patients with good periodontal health. Case: 30 patients with generalized stage III or IV periodontitis.	Periodontal disease	ELISA	-	Interleukin-33 (IL-33) ↑
Kluknavská et al., 2022 [44]	Case-control study	Number of subjects: 82 subjects. Control: 43 subjects with good periodontal health. Cases: (i) 23 subjects with chronic periodontitis, and (ii) 16 subjects with aggressive periodontitis.	Periodontitis	ELISA	-	MIP-1α ↑ Metalloproteinase-2 (MMP-2) ↑ Metalloproteinase-9 (MMP-9) ↑
Nair et al., 2022 [45]	Case-control study	Number of subjects: 90 individuals (45 male and 45 female subjects aged 20–50 years old). Control: Subjects with good gingival health. Cases: (i) Subjects with chronic gingivitis, and (ii) subjects with chronic periodontitis.	Periodontal disease	ELISA	IL-17 ↑ IL-18 ↑ IL-21 ↑	-
Reddahi et al., 2022 [31]	Cross-sectional study	Number of subjects: 40 randomly selected subjects (10 male and 30 female subjects aged 18 years old and above, non-smoker, with at least 20 teeth). Control: 10 subjects with good periodontal health. Case: 30 patients with periodontitis.	Periodontitis	Sandwich ELISA	-	Interleukin-1β (IL-1β) ↑ IL-6 ↑ Metalloproteinase (MMP-8) ↑
Salminen et al., 2022 [46]	Cross-sectional study	Number of subjects: 478 subjects (313 male and 165 female subjects with a mean age of 63 years old). Control: No mild periodontitis subjects. Case: Moderate–severe periodontitis subjects.	Periodontitis in elderly patients	ELISA and time-resolved immunofluorometric assay (IFMA)	-	S100A8 ↑ S100A12 ↑ Terminal complement complex (TCC) ↑ MMP-8 ↑
Aydin and Dilsiz, 2021 [47]	Case-control study	Number of subjects: 72 (39 male and 33 female subjects with mean age ranging from 30–37 years old). Control: 24 patients with healthy periodontal conditions. Cases: (i) 24 patients with gingivitis, and (ii) 24 patients with chronic periodontitis.	Periodontitis	ELISA	Oncostatin M (OSM) (GCF ↑ and saliva ↑) Interleukin-11 (IL-11) (GCF ↑ and saliva ↑)	
Baddam et al., 2021 [48]	Cross-sectional clinico-biochemical study	Number of subjects: 30 individuals (16 male and 14 female subjects with a mean age of 43 years old). Control: 15 subjects with good periodontal health. Case: 15 subjects with chronic periodontitis (age and gender matched).	Periodontitis	ELISA	Tartrate-resistant acid phosphatase (TRAP) ↑	-
Badros et al., 2021 [49]	Prospective observational study	Number of subjects: 110 multiple myeloma patients (68 male and 42 female patients with a mean age of 57 years old). Control: Patients with no BRONJ. Case: Patients who develop BRONJ.	Bisphosphonate-related osteonecrosis of the jaw	Luminex technology milliplex MAP kits	-	MIP-1β ↑ TNF-α ↑ IL-6 ↑
Görgün et al., 2021 [50]	Case-control study	Number of subjects: 95 subjects (35 male and 60 female subjects with a mean age of 28–35 years old). Control: 30 volunteers with good periodontal health. Cases: (i) 35 patients with generalized aggressive periodontitis, and (ii) 30 patients with generalized chronic periodontitis.	Periodontitis	ELISA	IL-1β ↑ IL-37 ↑	-
Joseph et al., 2020 [51]	Cross-sectional study	Number of subjects: 90 individuals (54 male and 36 female patients between the ages of 25 and 44 years old). Control: Healthy patients with good periodontal health. Cases: (i) Patients with generalized periodontitis stage I-III with non-smokers, and (ii) patients with generalized periodontitis stage I-III and current cigarette smokers.	Periodontal disease among smokers.	ELISA	-	OC ↑
Syed et al., 2020 [52]	Cross-sectional study	Number of subjects: 30 individuals (15 male and 15 female subjects with a mean age of 41 years old). Control: 15 subjects with good periodontal health. Case: 15 subjects with chronic periodontitis age and gender matched.	Periodontitis	ELISA	Deoxypyridinoline (DPD) ↑	-
Ansari Moghadam et al., 2019 [53]	Case-control study	Number of subjects: 27 subjects (13 male and 14 female subjects with mean age ranging from 33–37 years old). Control: 14 healthy controls. Case: 13 subjects with severe chronic periodontitis.	Severe chronic periodontitis	ELISA	-	RANKL/OPG ratio ↑
Batra et al., 2019 [54]	Cross-sectional study	Number of subjects: 90 patients comprising 54 males and 36 females from age 20–60 years old with at least 20 natural teeth (divided equally into three groups). Control: Patients with good periodontal health. Cases: (i) Patients with chronic periodontitis, and (ii) patients with aggressive periodontitis.	Periodontitis	ELISA	IL-34 ↑	-
Betsy et al., 2019 [55]	Cross-sectional study	Number of subjects: 90 patients, i.e., 53 males and 37 females with the age group 25–75 years old. Control: 30 patients with good periodontal health. Cases: (i) 30 patients with periodontitis without T2DM, and (ii) 30 patients with periodontitis and T2DM.	Periodontitis	ELISA	-	CTX ↑ OC ↑ ON ↑
Yilmaz et al., 2019 [56]	Case-control study	Number of subjects: 50 subjects Control: 10 subjects with good periodontal health. Cases: (i) 20 patients with chronic periodontitis, and (ii) 20 patients with generalized aggressive periodontitis.	Periodontitis	ELISA	MMP-3 ↑	-
Agrawal et al., 2018 [57]	Double-blind, case-control clinical study	Number of subjects: 80 female subjects with an age range of 35–55 years old. Controls: (i) 20 subjects of pre-menopausal women with good periodontal health, and (ii) 20 subjects of post-menopausal women with good periodontal health. Cases: (i) 20 subjects of pre-menopausal women with chronic periodontitis, and (ii) 20 subjects of post-menopausal women with chronic periodontitis.	Chronic periodontitis in post-menopausal women	ELISA	-	TNF-α ↑
Guruprasad and Pradeep, 2018 [58]	Case-control study	Number of subjects: 30 individuals (16 male and 14 female subjects aged between 30 and 56 years old). Control: 15 individuals with good periodontal health. Cases: (i) 15 individuals with chronic generalized periodontitis, and (ii) group III consisted of group II patients after 8 weeks of scaling and root planing treatment.	Periodontal disease	ELISA	IL-34 ↑	-
Lundmark et al., 2017 [59]	Case-control study	Number of subjects: 76 individuals (33 male and 43 female subjects) Control: 39 individuals who were healthy controls (mean age 37 years old). Case: 37 individuals with periodontitis (mean age of 62 years old).	Periodontitis	ELISA/Bradford assay	Mucin 4 (GCF ↓ and saliva ↓) MMP-7 (GCF ↑ and saliva ↑)	
Özden et al., 2017 [60]	Clinical trial study	Number of subjects: 47 post-menopausal women with a mean age of 55–57 years old. Controls: (i) 10 periodontally healthy individuals, and (ii) 12 periodontally healthy patients with osteoporosis. Cases: (i) 12 patients with chronic periodontitis, and (ii) 13 patients with both chronic periodontitis and osteoporosis.	Chronic periodontitis in post-menopausal osteoporosis patients	ELISA	OPG (GCF ↓ and saliva ↓)	
Schulze-Späte et al., 2017 [61]	Cross-sectional study	Number of subjects: 109 subjects consisting of 75 males and 34 females with a mean age of 52–57 years old. Control: 32 healthy controls. Cases: (i) 39 patients with heart failure, and (ii) 38 patients post-heart transplantation	Periodontitis among patients with heart disease	ELISA	IL-1β (GCF ↑ and saliva ↑) Β-glucuronidase (GCF ↑ and saliva ↑)	
Sophia et al., 2017 [62]	Case-control study	Number of subjects: 40 post-menopausal women aged between 45 and 60 years old with at least 20 teeth. Control: 20 post-menopausal women with good periodontal health. Case: 20 post-menopausal women with generalized chronic periodontitis.	Periodontitis among post-menopausal women	Beckman and Coulter, AU 480 auto analyser	-	ALP ↑
Ursarescu et al., 2016 [63]	Case-control study	Number of subjects: 38 patients with chronic periodontitis. Control: 18 systemically healthy patients with chronic periodontitis (mean age: 56 years old). Case: 20 patients with osteoporosis and chronic periodontitis (mean age: 55 years old).	Chronic periodontitis among osteoporotic patients	ELISA	IL-6 ↑ RANKL ↑	-
Aruna 2015 [64]	Clinico-biochemical study	Number of subjects: 30 subjects, i.e., 15 males and 15 females with an age range of 25–50 years old. Control: 10 subjects with good periodontal health Cases: (i) 10 subjects with gingivitis, (ii) 10 subjects with periodontitis, and (iii) patients after scaling and root planing.	Periodontal disease	Competitive ELISA	NTX ↑	
Elavarasu et al., 2015 [65]	Case-control study	Number of subjects: 30 subjects selected randomly. Control: 15 subjects with good periodontal health. Case: 15 subjects with chronic periodontitis.	Periodontal disease	ELISA	A disintegrin and metalloproteinase 8 (ADAM8) ↑	-
Hassan et al., 2015 [66]	Clinical trial study	Number of subjects: 30 subjects, i.e., 12 males and 18 females with an age range of 32–55 years old. Control: 10 periodontally healthy subjects. Case: 20 subjects with chronic periodontitis.	Chronic periodontitis	ELISA	OPG (GCF ↓ and saliva)	
Mishra et al., 2015 [67]	Case-control study	Number of subjects: 43 patients, i.e., 21 males and 22 females with an age range of 20–50 years old. Control: 11 healthy controls. Cases: (i) 17 patients with gingivitis, and (ii) 15 patients with periodontitis.	Periodontal disease	ELISA	-	Pyridinoline cross-linked carboxyterminal telopeptide of type I collagen (ICTP) ↑
Taichman et al., 2015 [68]	Case series study	Number of subjects: 58 post-menopausal women with a mean age of 61 years old for both groups. Control: 29 controls without breast cancer. Case: 29 subjects with breast cancer on aromatase inhibitors.	Periodontal health in women with early-stage post-menopausal breast cancer newly on aromatase inhibitors	Custom human array-based multiplex sandwich ELISA system	-	TNF-α ↑ OC ↑
Thumbigere-Math et al., 2015 [69]	Case-control study	Number of subjects: 40 subjects, i.e., 10 males and 30 females with a mean age of 62–64 years old. Control: 20 healthy controls. Case: 20 BRONJ patients.	Bisphosphonate-related osteonecrosis of the jaw (BRONJ)	ELISA	-	Matrix metalloproteinase-9 (MMP-9) ↑
Fine et al., 2014 [70]	Retrospective, longitudinal study	Number of subjects: 54 subjects. Control: 38 healthy subjects. Case: 16 subjects with *Aggregatibacter actinomycetemcomitans* and bone loss.	Aggressive periodontitis	Luminex/Millipore xMap system	Macrophage Inflammatory Protein (MIP-1α) (GCF ↑ and saliva ↑)	
Khongkhunthian et al., 2014 [71]	Cross-sectional study	Number of subjects: 53 subjects, i.e., 26 males and 27 females with a mean age of 26–50 years old. Control: 10 healthy volunteers. Cases: (i) 10 patients with gingivitis, and (ii) 33 patients with chronic periodontitis.	Periodontal disease	ELISA and fluorometric assay	Chondroitin sulfate ↑ Alkaline phosphatase ↑	-

## 3. Results

### 3.1. Data Analysis and Presentation

A total of 14,965 citations were retrieved from the five databases, while an additional 34 citations were found through website and grey literature (Google Scholar) searches, and by hand-searching references. After limiting the search to publications from the last 10 years, removing duplicates, and assessing the relevance of the articles based on the inclusion and exclusion criteria, the database search results were reduced to 5030 citations, and 26 citations remained from other sources. Following an eligibility assessment, 136 journal articles from the databases and 26 articles from other sources were included in the final stage. Ultimately, 37 articles were included in the review, as shown in Table 1. Most studies focused on biomarkers of periodontitis, followed by medication-related osteonecrosis of the jaw (MRONJ) or bisphosphonate-related osteonecrosis of the jaw (BRONJ), and osteoporosis.

### 3.2. Quality Assessment

Generally, the quality of all the selected articles was good, as they described most items clearly and comprehensively. The results of the quality assessment or risk of bias assessment are available in Supplementary S2. All studies showed a high risk for “confounders” and “blinding of assessors” items as they did not mention handling the confounders during the stages of planning or analysis [2,31,37,38,39,40,41,42,43,44,45,46,47,48,49,50,51,52,53,54,55,56,57,58,59,60,61,62,63,64,65,66,67,68,69,70,71]. Two studies presented a high risk for the “comparability of the target group” item as they did not indicate a clear statement on the selection of the comparison of target groups [43,47].

### 3.3. Bone Turnover Markers in GCF

Overall, 18 studies involving GCF were included in this review, with the majority focusing on periodontitis.

#### 3.3.1. Periodontal Disease

Two studies [39,40] showed that sclerostin levels were significantly higher in periodontitis patients compared to controls. As sclerostin is a protein that regulates osteoblast differentiation and proliferation, its elevated levels likely indicate active inhibition of bone formation [39,40]. Similarly, TRAP (tartrate-resistant acid phosphatase-5b or TRAcP-5b) is a specific marker of bone resorption in osteoclasts [72]. Increased levels of TRAP may suggest ongoing bone destruction [48]. Another marker of bone resorption, dihydropyrimidine dehydrogenase (DPD), was also studied. Elevated DPD levels in GCF indicate active disease progression and an increase in osteoclast activity in the bone [52].

In addition, numerous studies have examined inflammation markers such as interleukins, TNF-α, and matrix metalloproteinases (MMPs). These markers were significantly elevated in periodontitis patients, followed by gingivitis patients, and were lowest in healthy controls [45,50]. This increase was observed across different stages of periodontal disease [56].

#### 3.3.2. Medication-Related Osteonecrosis of the Jaw (MRONJ)

No studies on GCF biomarkers for MRONJ or BRONJ were identified in this review. In an animal model study, the pro-inflammatory marker IL-36α was found to be elevated in inflamed periodontal tissues [73]. However, no human GCF biomarkers related to bone turnover in MRONJ were reported [74].

#### 3.3.3. Osteoporosis

The continuous regulation of RANKL and OPG proteins is crucial for maintaining homeostasis in the bone remodeling process [53]. In a study by Özden et al., OPG levels increased in post-menopausal osteoporosis patients three to six months after receiving bisphosphonate (BP) treatment, likely due to the effect of BP in slowing bone loss [60]. Meanwhile, elevated GCF levels of IL-6 and RANKL were observed in patients with both chronic periodontitis and osteoporosis, compared to controls, indicating an active bone resorption process [63].

### 3.4. Bone Turnover Markers in Saliva

Overall, 24 studies used saliva samples to isolate and quantify specific bone turnover markers, with periodontitis being the most commonly studied disease.

#### 3.4.1. Periodontal Disease

Salivary CTX, OC, and ON have been identified as potential markers for periodontal disease [51,55]. These markers were found to be significantly elevated in periodontitis cases compared to controls [55], and their levels correlated with periodontal parameters such as Probing Pocket Depth (PPD), Bleeding on Probing (BOP), and bone loss (BL) [51,55]. Similar to the biomarkers found in gingival crevicular fluid (GCF), IL-1β has also been studied in saliva due to its association with bone resorption and inflammation [31,42,61]. Increased levels of salivary IL-1β, IL-6, and MMP-8 were observed in individuals with periodontitis [31]. Furthermore, the elevated ratio of the receptor activator of nuclear factor kappa-B ligand (RANKL) to osteoprotegerin (OPG) in saliva highlights its significant role in bone metabolism, which is consistent with findings from other studies [53]. Aydin and Dilsiz reported elevated levels of oncostatin M (OSM), leukemia inhibitory factor (LIF), and IL-11 in the saliva of patients with periodontitis and gingivitis, with levels decreasing following periodontal treatment [47]. Additionally, a study by Kluknavska et al. found that macrophage inflammatory protein-1α (MIP-1α), matrix metalloproteinase-2 (MMP-2), and MMP-9 levels were significantly higher in individuals with periodontal disease compared to controls [44].

#### 3.4.2. Medication-Related Osteonecrosis of the Jaw (MRONJ)

Unlike GCF, saliva has many potential biomarkers for MRONJ [75]. Various studies reported increased levels of inflammatory mediators such as MMP-9, TNF-α, and IL-6, which are also known to play an active role in periodontal bone loss [41,49,69]. Periodontal inflammation has been suggested as a potential risk factor for MRONJ [73].

#### 3.4.3. Osteoporosis

Increased levels of salivary BAP, OC, NTX, and CTX were observed in osteoporosis patients compared to healthy controls [2]. Additionally, elevated levels of calcium, OC, and ALP were detected in male osteoporotic patients [37]. In another study involving subjects with diabetes mellitus, ALP and BAP were found to have different roles, ALP was associated with vascular calcification, while BAP served as a marker for bone formation [76].

## 4. Discussion

Oral fluid and GCF contain disease mediators, including microbial agents, host-response markers, and bone-specific resorptive markers [27,77]. While there is extensive literature on oral fluid biomarkers, further investigation into GCF as a source of bone biomarkers is warranted. GCF contains abundant proteins and peptides that can be accurately analyzed using proteomic methods [77]. Potential biomarkers in GCF, such as proteins, cytokines, phosphatases, proteinases, and local tissue degradation products, highlight its promise for future studies on disease detection [78]. Since GCF is a component of saliva, biomarkers found in GCF are often detected in saliva as well, making saliva a more convenient sampling medium [79].

Table 2 below outlines the advantages and disadvantages of both fluids. Several studies agree that GCF and saliva are safe, non-invasive, easy to collect, and suitable for clinical testing [13,79,80]. However, some concerns exist regarding sample contamination with blood or dental plaque [13,81,82,83].

**Figure 2:**
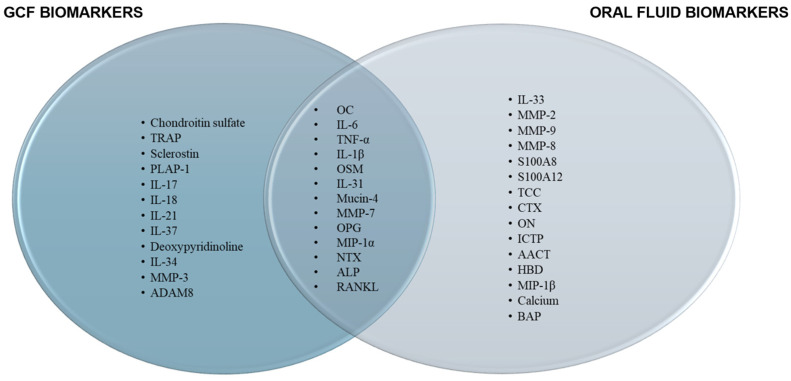
Biomarkers from the GCF and oral fluid.

Figure 2 presents a Venn diagram summarizing all biomarkers mentioned in the results in Section 3 for easier reference. Biomarkers studied in GCF primarily consist of inflammatory markers such as interleukins and TNF-α [40,47,50], in addition to bone-related markers such as OC, sclerostin, and CTX [2,39,55]. These biomarkers were detected in patients with both periodontal disease and osteoporosis. IL-1β was extensively studied in both GCF and saliva, based on the number of studies included in this review [31,42,61]. Additionally, the RANKL/OPG ratio [91] and matrix metalloproteinases (MMPs) [92], which are enzymes involved in bone turnover and extracellular matrix degradation, were identified as potential markers for bone resorption.

Although several biomarkers were detectable in both GCF and saliva, some were insignificant and lacked sufficient evidence for clinical application [75,93,94]. Among the salivary biomarkers, OC and CTX were the most frequently studied proteins [51,55,95]. Regarding pro-inflammatory cytokines, increased levels of IL-6 are associated with bone resorption by promoting osteoclast differentiation [31,75,96]. Other studies reported elevated levels of salivary BAP, OC, CTX, and NTX in osteoporosis, indicating rapid bone turnover [2,55]. Despite the identification of numerous biomarkers in both GCF and saliva, none were exclusive to the oral cavity; they were present systemically throughout the body, complicating the ability to make precise disease diagnoses based on oral fluid samples alone [31]. However, as systemic markers, these biomarkers can reflect an individual’s overall health status [97].

### 4.1. Role of Biomarkers in Bone Turnover

The bone formation markers identified in this study include OC and BAP [2] (Table 3). Serum OC is a well-established indicator of osteoblast activity in osteoporotic patients [98]. Originating from osteoblasts, OC binds to hydroxyapatite crystals to promote bone formation [99,100], while BAP enhances bone mineralization [101]. TRAcP-5b is an enzyme produced by osteoclasts, and CTX and NTX are byproducts of osteoclasts and type I collagen breakdown [98]. Deoxypyridinoline (DPD) stabilizes bone structure with type I collagen and is released during bone remodeling [98]. Calcium, in conjunction with parathyroid hormone (PTH), regulates bone calcium levels [102]. PTH reduces osteoblast activity and stimulates osteoclasts, leading to bone resorption and calcium release into the bloodstream [102]. Cross-linked telopeptide of type I collagen (ICTP) is a predominant collagen in alveolar bone, which is released into the blood during inflammation, making it a marker for bone resorption [67]. Regulators of bone turnover, such as RANKL and OPG, maintain the balance between bone resorption and formation. RANKL stimulates osteoclasts for bone resorption, while OPG inhibits it by binding to RANKL, preventing its interaction with the RANK receptor on osteoclasts [103]. Sclerostin, produced by osteocytes, inhibits osteoblast activity and bone formation [98,104].

In summary, most common bone turnover markers were present in this study, except for P1NP, bone sialoprotein (BSP), and osteopontin (OP). P1NP is produced by fibroblasts and osteoblasts during bone remodeling [98], while BSP and OP, located in the bone matrix, contribute to bone mineralization [105].

**Table 3:** General bone turnover markers.

Bone Formation Markers (Osteoblasts)	Bone Turnover Regulators	Bone Resorption Markers (Osteoclasts)
OC BAP P1NP *	RANKL OPG Sclerostin	CTX NTX TRAcP-5b DPD Calcium ICTP BSP and OP *

* Not present in this scoping review.

### 4.2. Crossroads between Periodontitis, MRONJ, and Osteoporosis

Periodontitis is increasingly recognized for its role in interacting with and exacerbating various inflammatory conditions, including medication-related osteonecrosis of the jaw (MRONJ) and osteoporosis [7,105,106]. In the microenvironment associated with MRONJ, periodontitis, and other bone diseases, bone tissue is subjected to oxidative stress, endotoxemia, and the release of numerous growth factors and inflammatory mediators, such as IL-1β, IL-6, and TNF-α, which are detectable in both human gingival crevicular fluid (GCF) and saliva [107,108,109]. Periodontal inflammation has been identified as a risk factor for MRONJ [7,73], and the biomarkers involved in periodontitis are also shared with MRONJ and osteoporosis.

To effectively detect bone turnover diseases of the jawbone, such as osteoporosis and MRONJ, it is essential to study the biomarkers of periodontitis in conjunction with disease-specific markers after treatment with bisphosphonate or other drugs.

### 4.3. Limitations of This Study

Diagnosing a disease, particularly complex diseases such as oral conditions or diseases, requires the identification of more than one biomarker for complete justification. A single biomarker is insufficient for clinical testing and analysis. This has made the journey of the identification of biomarkers challenging as it is difficult to prove the accuracy of one biomarker for a specific disease, let alone more than one biomarker. Most studies that used GCF in this review were mainly published on periodontitis, although oral fluid or salivary biomarkers are currently being extensively studied as described above. 

Although the analysis of GCF and saliva is often interpreted as indicative of localized body inflammation due to their site-specific origins in oral conditions [15,86], the other oral conditions or diseases have not been studied extensively.

### 4.4. Future Clinical Use

Biochemical markers of bone turnover are increasingly recognized as essential, non-invasive, and cost-effective tools for evaluating bone metabolism in population studies, with their integration into clinical practice gaining momentum [110]. While their main use has been in monitoring treatment response, there is an urgent need for research that enhances early diagnosis by integrating these markers with indicators of biological and pharmacological responses to therapy, as well as disease-specific and immune response parameters, including protein expression, genomics, and transcriptomics [110]. This comprehensive approach could significantly improve the precision and effectiveness of diagnostic strategies.

## 5. Conclusions

In this scoping review, the most significant GCF biomarkers identified were IL-1β, sclerostin, OPG, and IL-34, while the key oral fluid biomarkers included OC, IL-1β, TNF-α, IL-6, OPG, and MMP-9. Notably, many biomarkers were found to overlap between GCF and oral fluid. Given this overlap, it is important to identify other specific biomarkers related to osteoporosis and MRONJ to enable accurate diagnosis of these conditions.

## Figures and Tables

**Table 2:** Comparison of GCF and oral fluid collection.

GCF Collection	Oral Fluid Collection
**Advantages**	
Safe and non-invasive [13,79].	Safe and non-invasive [34].
Easy to collect compared to blood [13,79].	Easier to collect compared to blood and GCF [28,79].
Reduced risk of complications in contrast with the blood sampling method [84].	Reduced risk of complications in contrast with the blood sampling method [84].
Low-cost and biologically acceptable samples collected by patients and dentists to be used for analysis [13].	Cheaper and affordable compared to blood and serum testing [85].
The results are more promising compared to saliva collection as the location of GCF is closer to the subgingival area (site specific) [14,83,86].	Feasible for clinical testing [34].
Proteins such as cytokines, enzymes, and tissue discharge products are released in the GCF upon inflammation and can be measured using paper points [13].	Proteins in the blood can be detected in saliva [85].
GCF sample is thought to reflect the state of periodontal health [87].	An alternative for individuals with difficulty or fear of needles during blood taking [88].
**Disadvantages**	
The sample could be contaminated by blood [89].	The sample could be contaminated by blood [81].
The sample could be contaminated with saliva during the sample collection process [13,89].	Sample collection mirrors the whole mouth condition and is therefore influenced by other factors such as the oral microbiome factors [83].
The sample could be contaminated with dental plaque [89].	May be influenced by one’s medication [90].
Difficult to extract GCF from paper points and the sample is in minute amounts [86].	Proteolytic enzymes in saliva may destroy salivary proteins that are crucial for analysis [90].

## Data Availability

The data presented in this study are available upon request from the corresponding author.

## Supplementary Materials

The following supporting information can be downloaded at: https://www.mdpi.com/article/10.3390/diagnostics14192184/s1. Supplementary S1: SCOPING REVIEW KEYWORDS AND DATABASE SEARCH STRATEGY. Supplementary S2: Quality assessment using the RoBANS 2 scale.
