# Supplementary material for: Oral and Gingival Crevicular Fluid Biomarkers for Jawbone Turnover Diseases: A Scoping Review
Source: Diagnostics (Basel). 2024 Sep 30;14(19):2184. doi: 10.3390/diagnostics14192184 (PMC11475764; doi:10.3390/diagnostics14192184)
Supplement: Supplementary file 1 [file diagnostics-14-02184-s001.zip › diagnostics-3227244-supplementary.pdf]

Supplementary S1: SCOPING REVIEW KEYWORDS AND DATABASE SEARCH STRATEGY

**Main keywords**	**Definition**	**MeSH search**	**Synonym/ other names**
Biomarkers	*"A defined characteristic that is measured as an indicator of normal biological processes, pathogenic processes or responses to an exposure or intervention" (Califf, 2018)*	*Measurable and quantifiable biological parameters (e.g., specific enzyme concentration, specific hormone concentration, specific gene phenotype distribution in a population, presence of biological substances) which serve as indices for health- and physiology-related assessments, such as disease risk, psychiatric disorders, environmental exposure and its effects, disease diagnosis; metabolic processes; substance abuse; pregnancy; cell line development; epidemiologic studies; etc.*	Biological markers, biochemical markers, biological factors, clinical markers, molecular markers
Fluids and secretions	*"Fluid, in physiology, a water-based liquid that*	*Liquid substances produced by living organisms to fulfill*	Gingival crevicular fluid, saliva, bodily

	contains the ions and cells essential to body functions and transports the solutes and products of metabolism. *Secretion, in biology, production and release of a useful substance by a gland or cell; also, the substance produced" (Britannica website)*	*specific functions or excreted as waste.*	secretions, exudates and transudates
Bone diseases	*"Bone diseases can make your bones weaker than normal and even break and deform them. Osteoporosis, osteopaenia and Paget's disease of bone are some of the more common bone diseases" (Healthdirect website)*	*Diseases of BONES.*	Bisphosphonate related osteonecrosis of the jaw OR medication related osteonecrosis of the jaw OR osteoporosis OR periodontitis OR periodontal disease

DATABASE SEARCH STRATEGY:

Database 1: Web of Science (WoS) **Date & Time: 25/08/2024 (10:23 PM)**

**No.**	**Search string/strategy**	**No. of hits/ search results**
1.	Biomarker OR biological marker OR biochemical marker OR biological factor OR clinical marker OR molecular marker	1,327,168
2.	Oral fluid OR fluid and secretion OR gingival crevicular fluid OR saliva OR bodily secretion OR exudate and transudate	127,415
3.	Bone disease OR bisphosphonate related osteonecrosis of the jaw OR medication related osteonecrosis of the jaw OR osteoporosis OR periodontitis OR periodontal disease	495,100
4.	*#1 AND #2*	11,137
5.	*#1 AND #3*	39,877
6.	*#2 AND #3*	10,064
7.	*#1 AND #2 AND #3*	1,889

Database 2: EBSCOhost Dentistry & Oral Sciences Source

Date & Time: 25/08/2024 (10:49 PM)

**No.**	**Search string/strategy**	**No. of hits/ search results**
1.	Biomarker OR biological marker OR biochemical marker OR biological factor OR clinical marker OR molecular marker	21,495
2.	Oral fluid OR fluid and secretion OR gingival crevicular fluid OR saliva OR bodily secretion OR exudate and transudate	57,175
3.	Bone disease OR bisphosphonate related osteonecrosis of the jaw OR medication related osteonecrosis of the jaw OR osteoporosis OR periodontitis OR periodontal disease	98,929
4.	*#1 AND #2*	8,697
5.	*#1 AND #3*	11,078
6.	*#2 AND #3*	23,943
7.	*#1 AND #2 AND #3*	6,094

Database 3: Cochrane Library **Date & Time: 25/08/2024 (10:59 PM)**

**No.**	**Search string/strategy**	**No. of hits/ search results**
1.	Biomarker OR biological marker OR biochemical marker OR biological factor OR clinical marker OR molecular marker	39,061
2.	Oral fluid OR fluid and secretion OR gingival crevicular fluid OR saliva OR bodily secretion OR exudate and transudate	18,417
3.	Bone disease OR bisphosphonate related osteonecrosis of the jaw OR medication related osteonecrosis of the jaw OR osteoporosis OR periodontitis OR periodontal disease	43,984
4.	*#1 AND #2*	1,089
5.	*#1 AND #3*	2,552
6.	*#2 AND #3*	1,751
7.	*#1 AND #2 AND #3*	221

Database 4: Scopus **Date & Time: 25/08/2024 (11:06 PM)**

**No.**	**Search string/strategy**	**No. of hits/ search results**
1.	Biomarker OR biological marker OR biochemical marker OR biological factor OR clinical marker OR molecular marker	2,387,562
2.	Oral fluid OR fluid and secretion OR gingival crevicular fluid OR saliva OR bodily secretion OR exudate and transudate	77
3.	Bone disease OR bisphosphonate related osteonecrosis of the jaw OR medication related osteonecrosis of the jaw OR osteoporosis OR periodontitis OR periodontal disease	11,760
4.	*#1 AND #2*	25
5.	*#1 AND #3*	2,546
6.	*#2 AND #3*	1
7.	*#1 AND #2 AND #3*	1

Database 5: Pub Med **Date & Time: 25/08/2024 (11:11 PM)**

**No.**	**Search string/strategy**	**No. of hits/ search results**
1.	Biomarker OR biological marker OR biochemical marker OR biological factor OR clinical marker OR molecular marker	4,643,885
2.	Oral fluid OR fluid and secretion OR gingival crevicular fluid OR saliva OR bodily secretion OR exudate and transudate	631,371
3.	Bone disease OR bisphosphonate related osteonecrosis of the jaw OR medication related osteonecrosis of the jaw OR osteoporosis OR periodontitis OR periodontal disease	977,988
4.	*#1 AND #2*	170,072
5.	*#1 AND #3*	151,965
6.	*#2 AND #3*	17,342
7.	*#1 AND #2 AND #3*	6,760

Grey Literature: Google Scholar **Date & Time: 25/08/2024 (11:27 PM)**

No.	Search string/strategy	No. of hits/ search results
1.	Biomarker OR biological marker OR biochemical marker OR biological factor OR clinical marker OR molecular marker	4,960,000
2.	Oral fluid OR fluid and secretion OR gingival crevicular fluid OR saliva OR bodily secretion OR exudate and transudate	2,620
3.	Bone disease OR bisphosphonate related osteonecrosis of the jaw OR medication related osteonecrosis of the jaw OR osteoporosis OR periodontitis OR periodontal disease	41,400
4.	*#1 AND #2*	906
5.	*#1 AND #3*	7,900
6.	*#2 AND #3*	52
7.	*#1 AND #2 AND #3*	19

RESULTS OF LITERATURE SEARCH*

The final list of studies or references obtained from all five databases and grey literature after screening of titles, abstracts and research design:

**No.**	**Title of Journal Article**	**Year**	**Author**	**Journal name, volume, issue and page number**	**Is the paper relevant to the research? (Yes (Y)/ No (N))**	**Is the full paper available for free (Yes (Y)/ No (N))**
1.	*Salivary Interleukin-6 as a Non-Invasive Biomarker for Chronic Periodontitis and Tooth Loss in Type 2 Diabetes*	*2024*	*Sangappa et al.*	*Indian Journal of Dental Research 35(1)*	*Y*	*Y*
2.	*Evaluation of Biomarkers of Bone Metabolism on Salivary Matrix in the Remodeling of Periodontal Tissue during Orthodontic Treatment*	*2024*	*Cazzolla et al.*	*Dentistry Journal 12, 209*	*N*	*Y*
3.	*Evaluation of temporomandibular joint morphology and morphometry in male osteoporotic patients using advanced imaging and biochemical markers: A cross sectional study*	*2024*	*Kareem & Muhealdeen*	*Cellular and Molecular Biology 70(6), 155-163*	*Y*	*Y*
4.	*Assessment of sclerostin levels in the gingival crevicular fluid of patients with periodontitis: A clinico-biochemical crosssectional study*	*2023*	*Ashifa et al.*	*Journal of Advanced Periodontology & Implant Dentistry 15(1), 3-9*	*Y*	*Y*
5.	*The Impact of Apical Periodontitis and Endodontic Treatment on Salivary Inflammatory Biomarkers: A Longitudinal Study*	*2023*	*Bakhsh et al.*	*Applied Sciences 13, 3952*	*N*	*Y*
6.	*Bone Biomarkers Measured on Salivary Matrix: Study of Biological Variability in a Cohort of Young Subjects*	*2023*	*Brescia et al.*	*Applied Sciences 13; 10234.*	*N*	*Y*
7.	*Macrophage Inflammatory Protein-1a Shows Predictive Value as a Risk Marker for Subjects and Sites Vulnerable to Bone Loss in a Longitudinal Model of Aggressive Periodontitis*	*2023*	*Fragkioudakis et al.*	*Dentistry Journal 11, 61*	*N*	*Y*
8.	*Gingival crevicular fluid Periodontal Ligament-associated Protein-1, Sclerostin, and Tumor Necrosis Factor-alpha Levels in Periodontitis*	*2023*	*Gür et al.*	*Journal of Periodontology 94(10); 1166-1175*	*Y*	*Y*
9.	*Role of six cytokines and bone metabolism biomarkers in gingival crevicular fluid in patients undergoing fixed orthodontic appliance treatment in comparison with aligners: a clinical study*	*2023*	*Kamran et al.*	*Angle Orthodontist 93(3)*	*N*	*Y*
10.	*Assessment of Salivary Levels of the RANKL and RANK in Patients with Healthy Gingiva on Reduced Periodontium Versus Periodontitis: An Analytical Cross-Sectional Study*	*2023*	*Muhssin & Akram*	*Dental Hypotheses*	*N*	*Y*
11.	*Alpha-Defensin 1: An Emerging Periodontitis Biomarker*	*2023*	*Lee et al.*	*Diagnostics 13, 2143*	*N*	*Y*
12.	*Assessment of Bone Turnover Markers Prior to Dental Implant Placement for*	*2023*	*Nirubama et al.*	*Annals of Dental Specialty 11(2); 57-61*	*Y*	*Y*

	Osteoporosis Patient- A Case-Control Study					
13.	*Analysis of Salivary Levels of IL-1β, IL17A, OPG and RANK-L in Periodontitis Using the 2017 Classification of Periodontal Diseases—An Exploratory Observational Study*	*2023*	*Relvas et al.*	*Journal of Clinical Medicine 12, 1003*	*Y*	*Y*
14.	*Protein and mRNA expression of interleukin-33 in periodontally diseased and healthy individuals and impact of nonsurgical periodontal therapy in salivary IL-33 levels*	*2023*	*Renjith et al.*	*Journal of Indian Society of Periodontology*	*Y*	*Y*
15.	*A Study of Oral Health Parameters and the Properties and Composition of Saliva in Oncological Patients with and without Medication-Related Osteonecrosis of the Jaw Who Take Bisphosphonates*	*2023*	*Sobczak-Jaskow et al.*	*Medicina 59, 1073*	*N*	*Y*
16.	*Pleckstrin Levels Are Increased in Patients with Chronic Periodontitis and Regulated via the MAP Kinase-p38α Signaling Pathway in Gingival Fibroblasts*	*2022*	*Abdul Alim et al.*	*Frontiers in Immunology Vol. 12*	*N*	*Y*
17.	*Assessment of salivary alpha amylase and mucin-4 before and after non-surgical treatment of peri-implant mucositis*	*2022*	*Aldulaijan et al.*	*International Journal of Implant Dentistry*	*N*	*Y*
18.	*Comparison of periodontal status and salivary IL-15 and -18 levels in cigarette-smokers and individuals using electronic nicotine delivery systems*	*2022*	*Ali et al.*	*BMC Oral Health 22, 655*	*N*	*Y*
19.	*Risk factors for Medication-related Osteonecrosis of the Jaw and Salivary IL-6 in Cancer Patients*	*2022*	*Kemp et al.*	*Journal of Otorhinolaryngology 88(5); 683-690*	*N*	*Y*
20.	*Bone Turnover Markers in Gingival Crevicular Fluid and Blood Serum of Patients with Fixed Orthodontic Appliances*	*2022*	*Kloukos et al.*	*European Journal of Orthodontics 44(4); 412-419*	*N*	*Y*
21.	*Expression of Selected Inflammatory Proteins and Metalloproteinases in Periodontitis*	*2022*	*Kluknavská et al.*	*European Review for Medical and Pharmacological Sciences 26(6); 1825-1831*	*Y*	*Y*
22.	*Quantitative proteomics in medication-related osteonecrosis of the jaw: A proof-of-concept study*	*2022*	*Lorenzo-Pouso et al.*	*Oral Diseases 29(5), 2117-2129*	*Y*	*Y*
23.	*The Effect of Non-Surgical Periodontal Therapy on Pentraxin-3 Concentration in Gingival Crevicular Fluid of Chronic Periodontitis Patients: A Clinical Trial*	*2022*	*Mozaffari et al.*	*Journal of Dentistry (Shiraz, Iran) 23(3), 314-320*	*N*	*Y*
24.	*Comparative Evaluation of Gingival Crevicular Fluid Interleukin-17, 18 and 21 in Different Stages of Periodontal Health and Disease*	*2022*	*Nair et al.*	*Medicina 58, 1042*	*Y*	*Y*
25.	*Quantification of Salivary Nitric Oxide in Patients with Fixed Orthodontic Treatment*	*2022*	*Raducanu et al.*	*Applied Sciences 12, 8565*	*N*	*Y*
26.	*Salivary Biomarkers in Periodontitis Patients: A Pilot Study*	*2022*	*Reddahi et al.*	*International Journal of Dentistry 2022; 3664516*	*Y*	*Y*
27.	*Common complement factor H polymorphisms are linked with periodontitis in elderly patients*	*2022*	*Salminen et al.*	*Journal of Periodontology 93, 1626-1634*	*Y*	*Y*
28.	*Calprotectin and N-telopeptide of Type I Collagen (NTx) as Gingival Crevicular Fluid (GCF) Biomarker in Peri-Implantitis Patients*	*2022*	*Swarup et al.*	*Cureus 14(8)*	*N*	*Y*

29.	*Association between rheumatoid factors and proinflammatory biomarkers with implant health in rheumatoid arthritis patients with dental implants*	*2021*	*Alenazi*	*European Review for Medical and Pharmacological Sciences 25, 7014-7021*	*N*	*Y*
30.	*Measurement of Oncostatin M, Leukemia Inhibitory Factor, and Interleukin-11 Levels in Serum, Saliva, and Gingival Crevicular Fluid of Patients with Periodontal Diseases*	*2021*	*Aydin & Dilsiz*	*Meandros Medical and Dental Journal 22(3); 242-251*	*Y*	*Y*
31.	*Evaluation of Gingival Crevicular Fluid and Serum Tartrate-resistant Acid Phosphatase Levels in Subjects with Clinically Healthy Periodontium and Chronic Periodontitis – A Clinico-biochemical Study*	*2021*	*Baddam et al.*	*Journal of Pharmacy and Bioallied Sciences 13(Suppl 2), S1275-S1279*	*Y*	*Y*
32.	*Prospective Observational Study of Bisphosphonate-Related Osteonecrosis of the Jaw in Multiple Myeloma: Microbiota Profiling and Cytokine Expression*	*2021*	*Badros et al.*	*Frontiers in Oncology Vol. 11*	*Y*	*Y*
33.	*Monitoring Salivary Levels of Interleukin 1 Beta (IL-1β) and Vascular Endothelial Growth Factor (VEGF) for Two Years of Orthodontic Treatment: A Prospective Pilot Study*	*2021*	*Çevik-Aras et al.*	*Mediators of Inflammation Vol. 2021*	*N*	*Y*
34.	*Associations between Salivary Cytokines and Periodontal and Microbiological Parameters in Orthodontic Patients*	*2021*	*Chen et al.*	*Medicine 100(10); e24924*	*N*	*Y*
35.	*Effect of Non surgical Periodontal Treatment on Gingival Crevicular Fluid Interleukin-1beta and Interleukin-37 in Different Periodontal Diseases*	*2021*	*Görgün et al.*	*Meandros Medical and Dental Journal Vol. 22; 110-118*	*Y*	*Y*
36.	*Comparative Evaluation of Laser Biostimulation as an Adjunct to NSPT and Its Effects on AST Levels in the Management of Chronic Periodontitis: A Randomized Controlled Trial*	*2021*	*Gupta et al.*	*Journal of International Oral Health 13(3)*	*N*	*Y*
37.	*Biomarker levels in peri-implant crevicular fluid of healthy implants, untreated and non-surgically treated implants with peri-implantitis*	*2021*	*Hentenaar et al.*	*Journal of Clinical Periodontology*	*N*	*Y*
38.	*Usefulness of hemoglobin examination in gingival crevicular fluid during supportive periodontal therapy to diagnose the pre-symptomatic state in periodontal disease*	*2021*	*Ito et al.*	*Clinical Oral Investigations 25, 487-495*	*N*	*Y*
39.	*Comparative Evaluation of Sclerostin Levels in Gingival Crevicular Fluid in the Treatment of Chronic Periodontitis Patients Using Diode Laser as an Adjunct to Scaling and Root Planing: A Clinico-biochemical Study*	*2021*	*Pai et al.*	*Contemporary Clinical Dentistry*	*N*	*Y*
40.	*A Randomized, Phase 1, Placebo-Controlled Trial of APG-157 in Oral Cancer Demonstrates Systemic Absorption and An Inhibitory Effect on Cytokines and Tumor-associated Microbes*	*2020*	*Basak et al.*	*Cancer 126(8); 1668-1682*	*N*	*Y*
41.	*Salivary Osteocalcin as Potential Diagnostic Marker of Periodontal Bone Destruction among Smokers*	*2020*	*Joseph et al.*	*Biomolecules 10(3)*	*Y*	*Y*
42.	*Bone Turnover Markers in Serum but Not in Saliva Correlate with Bone Mineral Density*	*2020*	*Kerschan-Schindl et al.*	*Scientific Reports 10(1)*	*N*	*Y*
43.	*Evaluation of deoxypyridinoline levels in gingival crevicular fluid and serum as*	*2020*	*Syed et al.*	*Journal of Indian Society of Periodontology*	*Y*	*N*

	alveolar bone loss biomarker in patients with periodontitis			*24(4), 322-328*		
44.	*The Effect of Phase I Periodontal Treatment on the Salivary RANKL/OPG Ratio in Severe Chronic Periodontitis*	*2019*	*Ansari Moghadam et al.*	*Clinical Cosmetic and Investigational Dentistry Vol. 11; 251-257*	*Y*	*Y*
45.	*Comparative evaluation of Gingival Crevicular Fluid (GCF) levels of Interleukin-34 levels in periodontally healthy and in patients with chronic and aggressive periodontitis- A cross-sectional study*	*2019*	*Batra et al.*	*Saudi Dental Journal 31, 316-321*	*Y*	*Y*
46.	*Diagnostic Accuracy of Salivary Biomarkers of Bone Turnover in Identifying Patients with Periodontitis in a Saudi Arabian Population*	*2019*	*Betsy et al.*	*Journal of Dental Sciences 14(3); 269-276*	*Y*	*Y*
47.	*Sclerostin and WNT-5a gingival protein levels in chronic periodontitis and health*	*2019*	*Chatzopoulos et al.*	*Journal of Periodontal Research 54(5), 555-565*	*N*	*Y*
48.	*Periodontal disease, peri-implant disease and levels of salivary biomarkers IL-1β, IL-10, RANK, OPG, MMP-2, TGF-β and TNF-α: follow-up over 5 years*	*2019*	*Gomes et al.*	*Journal of Applied Oral Science 27*	*N*	*Y*
49.	*A Cross-sectional Cohort Study of Gingival Crevicular Fluid Biomarkers in Normal-weight and Obese Subjects during Orthodontic Treatment with Fixed Appliances*	*2019*	*Saloom et al.*	*Angle Orthodontist 89(6); 930-935*	*N*	*Y*
50.	*Metabolomic profiling reveals salivary hypotaurine as a potential early detection marker for medication-related osteonecrosis of the jaw*	*2019*	*Yatsuoka et al.*	*PLOS ONE*	*N*	*Y*
51.	*Pathogen profile and MMP-3 levels in areas with varied attachment loss in generalized aggressive and chronic periodontitis*	*2019*	*Yilmaz et al.*	*Central European Journal of Immunology 44(4)*	*Y*	*Y*
52.	*Estimation of Salivary Tumour Necrosis Factor-α Levels in Post-menopausal Women with Chronic Periodontitis*	*2018*	*Agrawal et al.*	*Journal of Clinical and Diagnostic Research 12(5)*	*Y*	*Y*
53.	*Evaluation of Chemokine CXCL10 in Human Gingival Crevicular Fluid, Saliva, and Serum as Periodontitis Biomarker*	*2018*	*Aldahlawi et al.*	*Journal of Inflammation Research Vol. 11; 389-396*	*N*	*Y*
54.	*Effect of Nonsurgical Periodontal Therapy on Interleukin-34 Levels in Periodontal Health and Disease*	*2018*	*Guruprasad and Pradeep*	*Indian Journal of Dental Research 29(3)*	*Y*	*Y*
55.	*Comparison of gingival crevicular fluid periostin levels in healthy, chronic periodontitis, and aggressive periodontitis*	*2018*	*Jamesha et al.*	*Journal of Indian Society of Periodontology 22(6), 480-486*	*N*	*Y*
56.	*Intraoral versus extraoral cementation of implant-supported single crowns: Clinical, biomarker, and microbiological comparisons*	*2018*	*Kiran et al.*	*Clinical Implant Dentistry and Related Research 20(2), 170-179*	*N*	*Y*
57.	*Magnetic Bead-based Salivary Peptidome Profiling for Accelerated Osteogenic Orthodontic Treatments*	*2018*	*Wu et al.*	*The Chinese Journal of Dental Research Vol. 21*	*N*	*Y*
58.	*The Effect of Scaling and Root Planning on Salivary TNF-α and IL-1α Concentrations in Patients with Chronic Periodontitis*	*2017*	*Eivazi et al.*	*Open Dentistry Journal Vol. 11, 573-580*	*N*	*Y*
59.	*Mucin 4 and matrix metalloproteinase 7 as novel salivary biomarkers for periodontitis*	*2017*	*Lundmark et al.*	*Journal of Clinical Periodontology 44, 247-254*	*Y*	*Y*

60.	*Effect of bisphosphonate as an adjunct treatment for chronic periodontitis on gingival crevicuar fluid levels of nuclear factor-κB ligand (RANKL) and osteoprotegerin in postmenopausal osteoporosis*	*2017*	*Özden et al.*	*Journal of Oral Science 59(1), 147-155*	*Y*	*Y*
61.	*Periodontitis and Bone Metabolism in Patients with Advanced Heart Failure and After Heart Transplantation*	*2017*	*Schulze-Späte et al.*	*ESC Heart Failure 4(2); 169-177*	*Y*	*Y*
62.	*Comparative Analysis of Salivary Alkaline Phosphatase in Post menopausal Women with and without Periodontitis*	*2017*	*Sophia et al.*	*Journal of Clinical and Diagnostic Research 11(1)*	*Y*	*Y*
63.	*Bone metabolism and RANKL/RANK/OPG trail in periodontal disease*	*2016*	*Czupkallo et al.*	*Current Issues in Pharmacy and Medical Sciences 29(3), 171-175*	*N*	*Y*
64.	*Evaluation of melatonin levels in saliva in gingivitis and periodontitis cases: A pilot study*	*2016*	*Lodhi et al.*	*Contemporary Clinical Dentistry 7(4)*	*N*	*Y*
65.	*Assessment of Bone Sialoprotein in the Saliva of Women at Peri- and Postmenopausal Age*	*2016*	*Piątek et al.*	*Journal of Pre-Clinical and Clinical Research 10(2); 100-104*	*N*	*Y*
66.	*Relation between the stability of dental implants and two biological markers during the healing period: a prospective clinical study*	*2016*	*Tirachaimongkol et al.*	*International Journal of Implant Dentistry 2, 27*	*N*	*Y*
67.	*The Assessment of IL-6 and Rankl in the Association Between Chronic Periodontitis and Osteoporosis*	*2016*	*Ursarescu et al.*	*Revista De Chimie 67(2)*	*Y*	*Y*
68.	*Estimation of N-terminal telopeptides of type I collagen in periodontal health, disease and after nonsurgical periodontal therapy in gingival crevicular fluid: A clinico - biochemical study*	*2015*	*Aruna*	*Indian Journal of Dental Research 26(2)*	*Y*	*Y*
69.	*Comparative analysis of gingival crevicular fluid a disintegrin and metalloproteinase 8 levels in health and periodontal disease: A clinic-biochemical study*	*2015*	*Elavarasu et al.*	*Journal of Pharmacy and Bioallied Sciences Vol. 7*	*Y*	*Y*
70.	*Effect of Periodontal Surgery on Osteoprotegerin Levels in Gingival Crevicular Fluid, Saliva, and Gingival Tissues of Chronic Periodontitis Patients*	*2015*	*Hassan et al.*	*Disease Markers 21(1), 46-56*	*Y*	*Y*
71.	*Evaluation of Salivary Levels of Pyridinoline Cross Linked Carboxyterminal Telopeptide of Type I Collagen (Ictp) in Periodontal Health and Disease*	*2015*	*Mishra et al.*	*Journal of Clinical and Diagnostic Research 9(9)*	*Y*	*Y*
72.	*Periodontal Health in Women with Early Stage Postmenopausal Breast Cancer Newly on Aromatase Inhibitors: A Pilot Study*	*2015*	*Taichman et al.*	*Journal of Periodontology 86(7), 906-916*	*Y*	*Y*
73.	*Salivary Proteomics in Bisphosphonate-Related Osteonecrosis of the Jaw*	*2015*	*Thumbigere-Math et al.*	*Oral Diseases*	*Y*	*Y*
74.	*Protein biomarkers and microbial profiles in peri-implantitis*	*2015*	*Wang et al.*	*Clinical Oral Implants Research 27, 1129-1136*	*N*	*Y*
75.	*Macrophage Inflammatory Protein-1a Shows Predictive Value as a Risk Marker for Subjects and Sites Vulnerable to Bone Loss in a Longitudinal Model of Aggressive Periodontitis*	*2014*	*Fine et al.*	*PLOS ONE 9(6)*	*Y*	*Y*

76.	*Comparisons between two biochemical markers in evaluating periodontal disease severity: a cross-sectional study*	*2014*	*Khongkhunthian et al.*	*BMC Oral Health 14, 107*	*Y*	*Y*
77.	*Clinical, microbiological, and salivary biomarker profiles of dental implant patients with type 2 diabetes*	*2014*	*Tatarakis et al.*	*Clinical Oral Implants Research 25, 803-812*	*N*	*Y*
78.	*Effect of periodontal therapy on lactoferrin levels in gingival crevicular fluid*	*2014*	*Yadav et al.*	*Australian Dental Journal 59, 314-320*	*N*	*Y*
REFERENCES/ LEGEND				***Journal articles obtained from five databases***		***Journal articles obtained from Grey Literature: Google Scholar***

***Journal articles final evaluation in Rayyan AI from August 2014- August 2024 (10 years)**

Supplementary S2: Quality assessment using the RoBANS 2 scale

Studies	Comparability of the target group	Target group selection	Confounders	Measurement of intervention/ exposure	Blinding of assessors	Outcome assessment	Incomplete outcome data	Selective outcome reporting
Mustafa et al., 2024 [38]	Low risk	Low risk	High risk	Low risk	High risk	Low risk	Low risk	Low risk
Sangappa et al., 2024 [39]	Low risk	Low risk	High risk	Low risk	High risk	Low risk	Low risk	Low risk
Ashifa et al., 2023[40]	Low risk	Low risk	High risk	Low risk	High risk	Low risk	Low risk	Low risk
Gür et al., 2023 [41]	Low risk	Low risk	High risk	Low risk	High risk	Low risk	Low risk	Low risk
Lorenzo-Pouso et al., 2023 [42]	Low risk	Low risk	High risk	Low risk	High risk	Low risk	Low risk	Low risk
Nirubama et al., 2023 [2]	Low risk	Low risk	High risk	Low risk	High risk	Low risk	Low risk	Low risk
Relvas et al., 2023 [43]	High risk	Low risk	High risk	Low risk	High risk	Low risk	Low risk	Low risk
Renjith et al., 2023 [44]	Low risk	Low risk	High risk	Low risk	High risk	Low risk	Low risk	Low risk
Kluknavská et al., 2022 [45]	Low risk	Low risk	High risk	Low risk	High risk	Low risk	Low risk	Low risk
Nair et al., 2022[46]	Low risk	Low risk	High risk	Low risk	High risk	Low risk	Low risk	Low risk
Reddahi et al., 2022 [32]	Low risk	Low risk	High risk	Low risk	High risk	Low risk	Low risk	Low risk
Salminen et al., 2022 [47]	High risk	Low risk	High risk	Low risk	High risk	Low risk	Low risk	Low risk
Aydin & Dilsiz, 2021 [48]	Low risk	Low risk	High risk	Low risk	High risk	Low risk	Low risk	Low risk
Baddam et al., 2021 [49]	Low risk	Low risk	High risk	Low risk	High risk	Low risk	Low risk	Low risk
Badros et al., 2021 [50]	Low risk	Low risk	High risk	Low risk	High risk	Low risk	Low risk	Low risk

Görgün et al., 2021 [51]	Low risk	Low risk	High risk	Low risk	High risk	Low risk	Low risk	Low risk
Joseph et al., 2020 [52]	Low risk	Low risk	High risk	Low risk	High risk	Low risk	Low risk	Low risk
Syed et al., 2020 [53]	Low risk	Low risk	High risk	Low risk	High risk	Low risk	Low risk	Low risk
Moghadam et al., 2019 [54]	Low risk	Low risk	High risk	Low risk	High risk	Low risk	Low risk	High risk
Batra et al., 2019 [55]	Low risk	Low risk	High risk	Low risk	High risk	Low risk	Low risk	Low risk
Betsy et al., 2019 [56]	Low risk	Low risk	High risk	Low risk	High risk	Low risk	Low risk	Low risk
Yilmaz et al., 2019 [57]	Low risk	Low risk	Low risk	Low risk	High risk	Low risk	Low risk	Low risk
Agrawal et al., 2018 [58]	Low risk	Low risk	High risk	Low risk	High risk	Low risk	Low risk	Low risk
Guruprasad & Pradeep, 2018[59]	Low risk	Low risk	High risk	Low risk	High risk	Low risk	Low risk	Low risk
Lundmark et al., 2017 [60]	Low risk	Low risk	High risk	Low risk	High risk	Low risk	Low risk	Low risk
Özden et al., 2017 [61]	Low risk	Low risk	High risk	Low risk	High risk	Low risk	Low risk	Low risk
Schulze-Späte et al., 2017 [62]	Low risk	Low risk	High risk	Low risk	High risk	Low risk	Low risk	Low risk
Sophia et al., 2017 [63]	Low risk	Low risk	High risk	Low risk	High risk	Low risk	Low risk	Low risk
Ursarescu et al., 2016 [64]	Low risk	Low risk	High risk	Low risk	High risk	Low risk	Low risk	Low risk
Aruna, 2015 [65]	Low risk	Low risk	High risk	Low risk	High risk	Low risk	Low risk	Low risk

Elavarasu et al., 2015 [66]	Low risk	Low risk	High risk	Low risk	High risk	Low risk	Low risk	Low risk
Hassan et al., 2015 [67]	Low risk	Low risk	High risk	Low risk	High risk	Low risk	Low risk	Low risk
Mishra et al., 2015 [68]	Low risk	Low risk	High risk	Low risk	High risk	Low risk	Low risk	Low risk
Taichman et al., 2015 [69]	Low risk	Low risk	High risk	Low risk	High risk	Low risk	Low risk	Low risk
Thumbigere-Math et al., 2015 [70]	Low risk	Low risk	High risk	Low risk	High risk	Low risk	Low risk	Low risk
Fine et al., 2014 [71]	Low risk	Low risk	High risk	Low risk	High risk	Low risk	Low risk	Low risk
Khongkhunthian et al., 2014 [72]	Low risk	Low risk	High risk	Low risk	High risk	Low risk	Low risk	Low risk
